# A randomized clinical trial evaluating the short-term results of ureteral stent encrustation in urolithiasis patients undergoing ureteroscopy: micro-computed tomography evaluation

**DOI:** 10.1038/s41598-021-89808-x

**Published:** 2021-05-14

**Authors:** Takashi Yoshida, Kuniko Takemoto, Yoshiko Sakata, Tomoaki Matsuzaki, Yuya Koito, Shimpei Yamashita, Isao Hara, Hidefumi Kinoshita, Tadashi Matsuda

**Affiliations:** 1 Department of Urology and Andrology, Kansai Medical University, 2‐5‐1, Shin‐machi, Hirakata, 573‐1152 Japan; 2 Department of Urology and Andrology, Kori Hospital, Kansai Medical University, Osaka, Japan; 3 Department of Physics, Kansai Medical University, Osaka, Japan; 4 Central Research of Laboratory, Kansai Medical University, Osaka, Japan; 5 Department of Urology, Wakayama Medical University, Wakayama, Japan

**Keywords:** Diseases, Medical research

## Abstract

Although many ureteral stents are commercially available, the actuality of encrustation is yet to be elucidated in humans. This study compared the Tria Ureteral Stent with PercuShield and the Polaris Ultra Ureteral Stent with HydroPlus Coating for short-term encrustation formation. Eighty-four patients, who required ureteral stent placement after ureteroscopy, were randomized into two stent groups. After stent removal on postoperative day 14, the encrustation volume on the stent surface was measured by micro-computed tomography. The primary outcome was the inner luminal encrustation volume. Secondary outcomes were encrustation volume on the outer or total surfaces and occurrence of adverse events. Clinical factors related to encrustation were also assessed as a post-hoc analysis. Finally, of the 82 patients analyzed, 75 (91.5%) had encrustation in the inner lumen of the stent. The difference in median inner encrustation volume between the Tria and Polaris Ultra stents was comparable (0.56 vs. 0.37 mm^3^, *P* = 0.183). There was no difference observed in the encrustation volume on the outer/total surfaces and stent-related adverse events. In both ureteral stents, the shaft body showed significant inner luminal encrustation compared to the proximal or distal loop (all, *P* < 0.05). Dyslipidemia (*P* = 0.027), elevated urine pH (*P* = 0.046), and crystalluria (*P* = 0.010) were associated with encrustation formation. The Tria and Polaris Ultra stents had similar efficacy for preventing encrustation in the short-term. Further studies are required to compare their long-term patency.

## Introduction

Ureteral stents are an essential tool in urologic practice, enabling decompression of ureteral obstruction, passive ureteral dilation, and preventing occlusion following endoscopic procedures^1^. However, stent placement is associated with discomfort, urinary infection, and encrustation^1^. Encrustation occurs when mineral crystals deposit on the inner and outer surfaces of an indwelling ureteral stent, leading to urinary infection, ureteral trauma, and difficulty in stent removal from the urinary tract. This phenomenon is observed even for short indwelling times (1–2 weeks) after implantation^2–4^. Therefore, many investigators and companies have focused on developing materials and coatings for ureteral stents, which can minimize encrustation^5^.

Previous studies investigating the efficacy of such materials or coatings were mostly performed using animal models or in vitro experiments with an artificial urine solution, but little is known about encrustation formation in humans^6^. To date, there has been almost no head-to-head clinical comparison of ureteral stents in reducing encrustation^5^. For assessing encrustation, electron microscopy and/or infrared spectroscopy are generally used to obtain a high-resolution image and evaluate surface coverage or chemical substances^4,7,8^. However, the techniques require that a ureteral stent is cut in parallel to observe the inner surface, and the encrusting material is dissolved to measure the amount of calcium and magnesium salts^4,7,8^. These complicated procedures create difficulties in quantifying the amount of encrustation objectively and conducting clinical trials that require a large number of stent samples.

In this randomized control trial, we compared two ureteral stents (Tria Ureteral Stent with PercuShield and the Polaris Ultra Ureteral Stent with HydroPlus) produced by different concepts of encrustation prevention; the former has a hydrophobic super smooth surface (new product), and the latter has a conventional hydrogel-coated surface. For analyzing encrustation, we were the first to use micro-computed tomography (micro-CT), a non-destructive method to visualize the fine internal structure of objects by capturing the differential absorption of the X-rays related to the local density of the material^9^ to measure encrustation volume on the inner and outer stent surfaces. This study aimed to investigate the superiority of the Tria ureteral stent versus the Polaris Ultra ureteral stent in preventing encrustation for the short-term indwelling time.

## Results

### Study population

**Figure 1:**
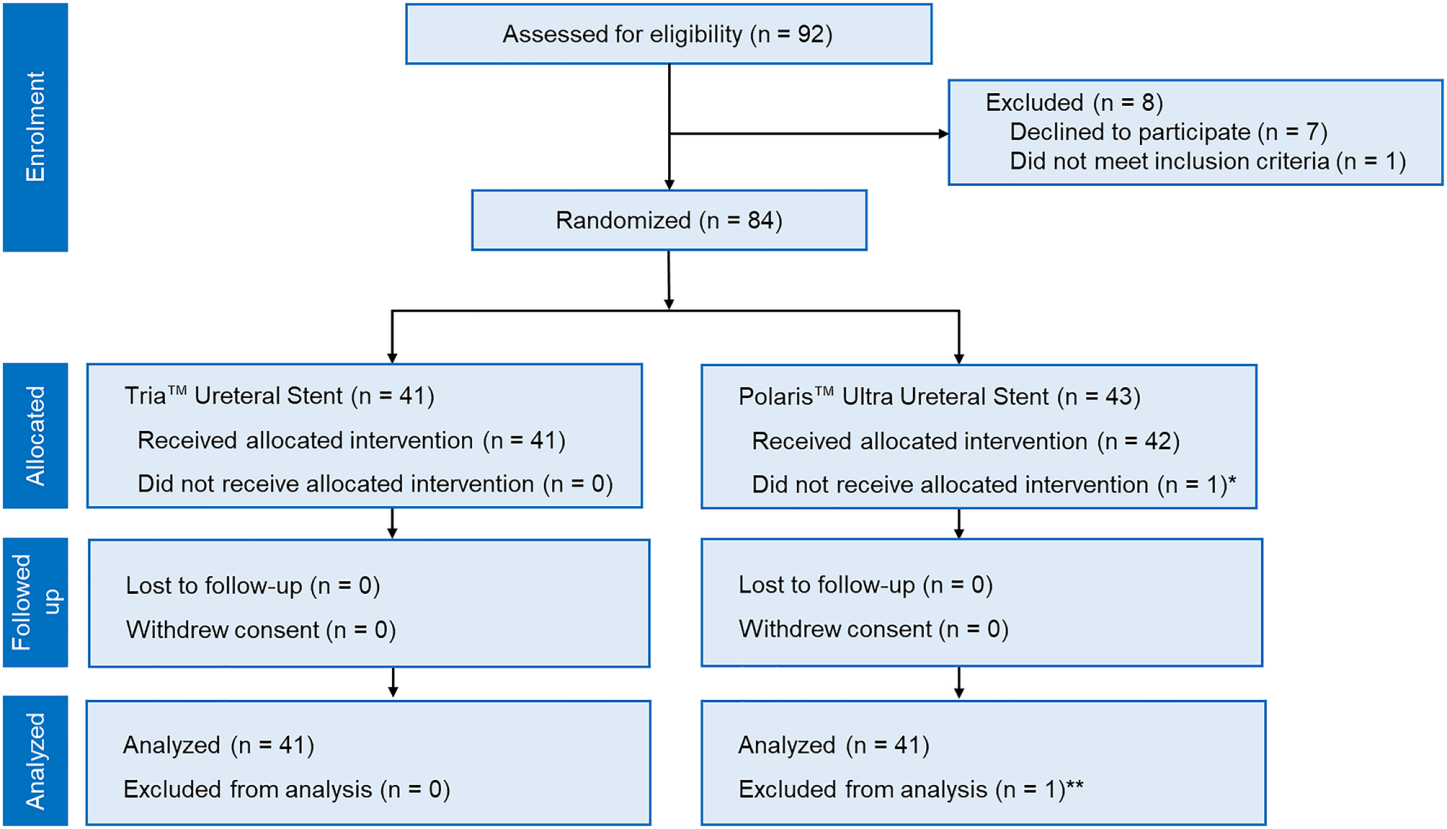
CONSORT diagram of the study. The symbol ^*^Indicates that surgery was not performed due to anaphylaxis. The symbol ^**^Indicates that the stent was discarded accidentally.

The CONSORT diagram of this study is shown in Fig. 1. In total, 84 patients were randomly assigned to two ureteral stent placement groups (ITT population) from July 2019 to November 2019. In the Polaris Ultra group, one patient did not undergo URS due to drug-induced anaphylaxis, and one was excluded from the final analysis because his ureteral stent was accidentally discarded before imaging evaluation. Finally, 82 patients were analyzed as a modified ITT population (Tria group, n = 41; Polaris Ultra group, n = 41).

The baseline characteristics of this cohort are shown in Table 1. All clinical variables were well-balanced between the two groups (all *P* > 0.05). Specifically, there was no significant difference in bacteriuria and positive urinary culture (*P* = 1.00 and 0.239, respectively).

**Table 1:** Baseline characteristics of the study cohort.

Variable	Tria ureteral stent	Polaris Ultra ureteral stent	*P*-value
	(n = 41)	(n = 43)	
**Age, year, median (IQR)**	62 (53–71)	69 (58–72)	0.339
**Sex, n (%)**			0.654
Female	17 (41.5)	15 (34.9)	
Male	24 (58.5)	28 (65.1)	
**Body mass index**, kg/m^2^, median (IQR)	25.01 (22.88–27.85)	23.32 (21.01–26.00)	0.142
**Comorbidities, n (%)**			
Hypertension	17 (41.5)	15 (34.9)	0.654
Dyslipidemia	10 (24.4)	5 (11.6)	0.160
Hyperuricemia	5 (12.2)	5 (11.6)	1.000
Diabetes mellitus	2 (4.9)	5 (11.6)	0.434
**Urine Analysis, n (%)**			
pH, median (IQR)	6.00 (5.50–6.50)	6.00 (5.50–6.50)	0.642
Hematuria	30 (73.2)	26 (60.5)	0.253
Pyuria	16 (39.0)	22 (51.2)	0.282
Bacteriuria	8 (19.5)	9 (20.9)	1.000
Crystalluria*	8 (19.5)	8 (18.6)	1.000
**Urinary culture**			0.239
Negative	8 (19.5)	15 (34.9)	
Positive**	6 (14.6)	7 (16.3)	
Missing	27 (65.9)	21 (48.8)	
**Hydronephrosis, n (%)**			0.924
Grade 0	17 (41.5)	16 (37.2)	
Grade 1	5 (12.2)	8 (18.6)	
Grade 2	11 (26.8)	11 (25.6)	
Grade 3	6 (14.6)	7 (16.3)	
Grade 4	2 (4.9)	1 (2.3)	
**Stone side, n (%**)			1.000
Left	16 (39.0)	17 (39.5)	
Right	25 (61.0)	26 (60.5)	
**Stone location, n (%)**			0.935
Renal pelvis	18 (43.9)	17 (39.5)	
Upper ureter	14 (34.1)	15 (34.9)	
Middle ureter	2 (4.9)	5 (11.6)	
Distal ureter	7 (17.1)	6 (14.0)	
**Stone size, mm, median (IQR)**	8.00 (6.04–10.00)	9.00 (6.72–11.00)	0.552

*IQR* interquartile range. *Calcium oxalate (n = 12), uric acid (n = 2**),** both (n = 1), and calcium phosphate (n = 1).

**Coagulase-negative *Staphylococcus* (n = 1), *Escherichia coli* (n = 2), *Proteus spp.* (n = 2), *Enterococcus spp*. (n = 4), *Klebsiella pneumoniae* (n = 2), and *Enterobacter* spp. (n = 2). Chi-squared test and Wilcoxon test were used for statistical analysis.

### Intra- and postoperative clinical data

The two groups showed no significant differences in the stone-free status, degree of hydronephrosis, incidence of specific stent-related complications, calls or visits to the medical centers, or duration of stent placement (all *P* > 0.05) (Table 2). The distribution of the stone components was also similar between the two groups (*P* = 0.51, Supplementary Fig. 1).

**Table 2:** Intra- and postoperative clinical data.

Variable	Tria Ureteral Stent	Polaris Ultra Ureteral Stent	*P*-value
	(n = 41)	(n = 42)	
**Surgical types**			0.697
Semi-rigid ureteroscopy	8 (19.5)	11 (26.2)	
Flexible ureteroscopy	31 (75.6)	30 (71.4)	
Combined	2 (4.9)	1 (2.4)	
**Ureteral access sheath use**	31 (75.6)	31 (73.8)	1.000
**Stone-free status at the surgery**	34 (82.9)	34 (81.0)	1.000
**Postoperative antibiotic use**	7 (17.1)	8 (19.0)	1.000
**Duration, day, median (range)**	0.00 (0.00–3.00)	0.00 (0.00–5.00)	0.493
**Postoperative complications**	2 (4.9)	3 (7.1)	1.000
Phone calls	3 (7.5)	1 (2.4)	0.353
Emergency room visit	3 (7.5)	1 (2.4)	0.353
Readmission	1 (2.5)	1 (2.4)	1.000
Urine Analysis*, n (%)			
pH, median (IQR)	6.00 (5.50–6.50)	6.00 (6.00–6.50)	0.291
Hematuria	35 (85.4)	36 (83.7)	0.349
Pyuria	24 (58.5)	26 (63.4)	0.500
Bacteriuria	11 (26.8)	10 (23.3)	0.296
Crystalluria	4 (9.8)	4 (9.3)	0.745
**Hydronephrosis, n (%)**			0.672
Grade 0	34 (82.9)	37 (90.2)	
Grade 1	4 (9.8)	3 (7.3)	
Grade 2	2 (4.9)	1 (2.4)	
Missing	1 (2.4)	0 (0.0)	
**Duration of stent placement, day, median (IQR)**	18.0 (15.0–18.0)	17.0 (15.0–18.0)	0.318

*IQR* interquartile range. Chi-square test and Wilcoxon test were used for statistical analysis.

*Six patients (Tria: n = 2, and Ultra: n = 4) were missing.

### Encrustation evaluated by micro-CT

Table 3 lists the mean encrustation volume of the total or segmental ureteral stent. Regarding the primary endpoint, there was no statistical difference in the inner luminal volume between the Tria and Polaris Ultra groups (0.56 vs. 0.37 mm^3^, *P* = 0.183). Further, no significant differences were found in the outer/total surfaces and segmental encrustation volume in each stent portion between the two groups (all *P* > 0.05).

**Table 3:** Comparison of encrustation volume between the two stents evaluated by micro-computed tomography.

	Tria Ureteral Stent	Polaris Ultra Ureteral Stent	*P*-value
	(n = 41)	(n = 41)	
**Overall encrustation volume, mm**^**3**^,** median (IQR)**			
Inner	0.56 (0.24–1.12)	0.37 (0.12–0.81)	0.183
Outer	0.00 (0.00–0.00)	0.00 (0.00–0.00)	0.663
Inner and outer	0.60 (0.24–1.29)	0.37 (0.12–0.90)	0.180
**Encrustation volume in proximal loop, mm**^**3**^,** median (IQR)**			
Inner	0.10 (0.00–0.45)	0.04 (0.00–0.23)	0.316
Outer	0.00 (0.00–0.00)	0.00 (0.00–0.00)	0.317
Inner and outer	0.10 (0.00–0.45)	0.04 (0.00–0.23)	0.316
**Encrustation volume in shaft body, mm**^**3**^,** median (IQR)**			
Inner	0.17 (0.04–0.57)	0.21 (0.01–0.37)	0.399
Outer	0.00 (0.00–0.00)	0.00 (0.00–0.00)	1.000
Inner and outer	0.18 (0.04–0.60)	0.22 (0.02–0.39)	0.437
**Encrustation volume in distal loop, mm**^**3**^,** median (IQR)**			
Inner	0.07 (0.00–0.22)	0.02 (0.00–0.10)	0.051
Outer	0.00 (0.00–0.00)	0.00 (0.00–0.00)	0.578
Inner and outer	0.07 (0.00–0.23)	0.02 (0.00–0.10)	0.091

*IQR* interquartile range. Chi-square test and Wilcoxon test were used for statistical analysis.

**Figure 2:**
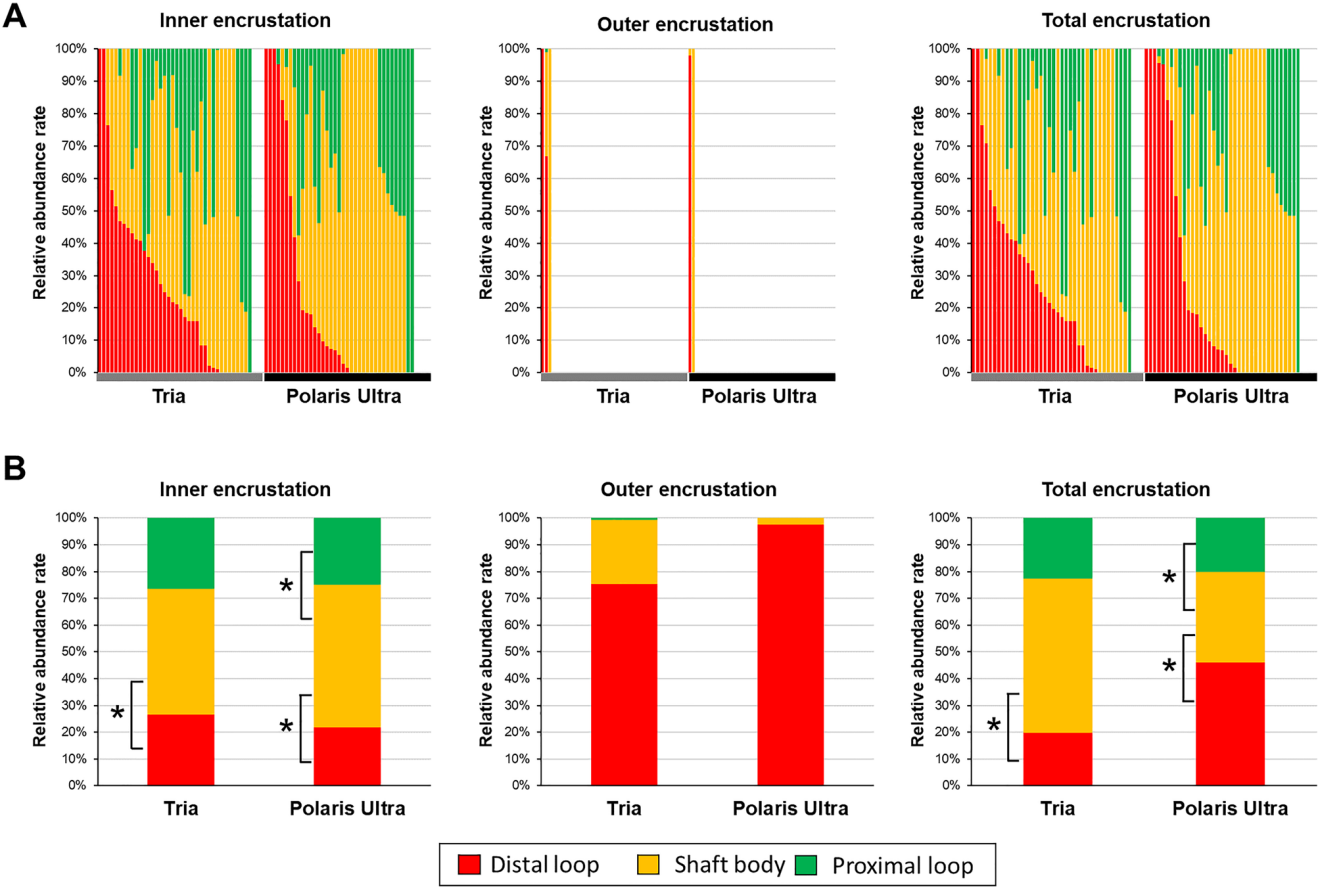
The relative abundance of encrustation at each stent portion (proximal loop/shaft body/distal loop) in each patient (**A**), and the average of each group (**B**). Height color bars demonstrate the percentage of encrustation volume at each portion, and the case with no bar displayed had no stent encrustation. The Friedman test with the Bonferroni correction was used for statistical analysis. *P < 0.05.

Figure 2A shows the relative abundance rate of encrustation at each stent portion in each patient, demonstrating that the encrustation in the inner lumen was seen in 75 (91.5%) patients (Tria: n = 38, Polaris Ultra: n = 37), and the outer surface encrustation was observed in only 5 (6.1%) patients (Tria: n = 3, Polaris Ultra: n = 2). Regarding the relative abundance rate of encrustation in the inner and total stent, the shaft body had a higher rate than the distal loop in the Tria group (*P* = 0.010 and *P* = 0.023, respectively), and the shaft body had higher rates compared to the proximal and distal loops in the Polaris Ultra group (inner: *P* = 0.006 and *P* = 0.009, respectively; and total: *P* = 0.003 and *P* = 0.031, respectively) (Fig. 2B). Although statistical analysis could not be performed due to the small sample size having encrustation, outer stent encrustation tended to occur in the distal loop in both groups (Fig. 2B).

### Associated factors with encrustation formation

Post hoc exploratory analyses were performed to identify the preoperative clinical factors related to the tendency of the total encrustation on the ureteral stent in the short-term period (Supplementary Table 1). The univariate analysis showed that patients with higher encrustation were more likely to have dyslipidemia (*P* = 0.040) and the presence of urine crystals (*P* = 0.048). After adjusting age, sex, and statistically relevant factors (*P*-value of < 0.1 in the univariate analysis), dyslipidemia (*P* = 0.027), higher urine pH (*P* = 0.046), and crystalluria (*P* = 0.010) were identified as independent risk factors for encrustation.

## Discussion

Although the design and technology of ureteral stents have advanced, and many products are commercially available, the actual functions and efficacy in preventing encrustation have not been clarified in a clinical setting^6^. The most common strategy for the prevention of encrustation is the use of a hydrophilic gel coating on stent surfaces, which accumulates water in its polymer network and acts as a deferent to hydrophobic bacterial surfaces and crystal deposits in the urine (e.g., Polaris Ultra stent is covered with Hydroplus )^10^. However, the Tria ureteral stent has been developed with a unique concept, PercuShield technology, in reducing urine crystal adhesion; the stent features nonionic, super smooth, hydrophobic inner lumen and outer surface processing against calcium and magnesium salt adhesion. Based on the company's study data on the bench-top test with an indwelling time of 2 weeks, the Tria (n = 15) showed a significant reduction in encrustation compared to the competitor stent (n = 15) in artificial sterile urine and *Proteus mirabilis* infected urine (www.bostonscientific.com/Tria).

However, this study showed that the Tria ureteral stent was comparable to the Polaris Ultra ureteral stent in reducing encrustation formation in the 2-week indwelling time, in contrast to the in vitro research results. There are several possible explanations for this result. First, the difference between the environment around the stent and in vitro setting (the concentration of minerals or crystals, urine flow dynamics, ureter peristalsis, the presence of protein, and types of bacteria) could affect the result of this study^11–13^. Second, the indwelling time of 2 weeks may be short for a significant difference between the two stent groups. Therefore, further studies with a relatively longer indwelling period that includes the loss of the hydrophilic coating may be required^14^. Furthermore, significant encrustation was seen in the inner lumen but not the outer surface in almost all patients, suggesting that contact with the ureteral wall, ureteral orifice, or urethral wall during stent removal may be associated with encrustation volume reduction on the outer surface. Thus, it may be reasonable to apply the inner encrustation volume as a primary endpoint in a short-term clinical trial.

Studies show that the key risk factors for the development of encrustation are the duration of stent indwelling time, bacterial biofilm, elevated urinary pH, and patient-specific factors^5,15^. Our study showed that higher urine pH and crystalluria were independent factors for encrustation formation, similar to previous reports^5,15^. However, there was no significant difference in bacteriuria and positive urinary culture. A possible explanation for this finding is that the use of intra- and post-operative antibiotics may restrict bacterial biofilm formation^16^. Further, we found an association between dyslipidemia and ureteral stent encrustation, a finding that has not been reported previously and could be attributed to the same mechanisms of urolithiasis formation. High triglycerides and total cholesterol caused increased urinary excretion of lithogenic components, such as oxalate, calcium, potassium, sodium, and chloride. Low high-density lipoprotein increased the urinary excretion of protective factors for stone formation, including citrate and magnesium^17,18^. Therefore, these risk factors should be considered in clinical decision-making to identify patients who need to avoid stent placement or shorten the indwelling time.

Our study has some limitations. First, the study design was a randomized, single-blind, multicenter trial. Hence, the possibility of technique bias in surgery cannot be fully excluded. Second, we did not evaluate 24-h urine compositions, such as oxalate, calcium, potassium, and sodium. Alternatively, we used a urinary sediment examination to evaluate crystals, which can be routinely obtained in daily practice. This trial was not powered for the reduction of encrustation-related complications as an endpoint. Finally, we did not evaluate ureteral stent discoloration because a study by Chew et al. suggested that stent discoloration did not result in an increased level of encrustation^7^.

## Conclusion

This randomized controlled trial indicated that the Tria ureteral stent and the Polaris Ultra ureteral stent had similar efficacy for preventing encrustation in the short-term period. To develop an “ideal ureteral stent” that minimizes encrustation, further research to understand the pathophysiology of encrustation and high-quality comparative studies are necessary.

## Material and methods

### Ethics statements

This study was a prospective, randomized, single-blinded (for participants), multicenter trial involving three academic centers: Kansai Medical University Hospital, Kansai Medical University Kori Hospital, and Wakayama Medical University Hospital. The study was registered at the University Hospital Medical Information Network (Registration No. UMIN000037006) on 20 June 2019 and approved by the ethics board of Kansai Medical University (IRB No. 2019026) on 24 June 2019. All patients provided written informed consent. We strictly followed the 2010 CONSORT statement guidelines to design and report this trial^19^.

### Study protocol

Adult patients (≥ 20 years old) who underwent unilateral ureteroscopy (URS) with planned ureteral stent insertion were eligible for this study. The exclusion criteria were as follows: (1) potential requirement for a ureteric stent for > 14 days postoperatively (e.g., patients with ureteral stricture); (2) active urinary infection; (3) current pregnancy or breastfeeding; (4) difficulty in obtaining consent. From July 2019 to November 2019, eligible patients were randomly divided into the Tria group and the Polaris Ultra group (1:1 ratio). After obtaining baseline clinical data, imaging for the stone, and urinary sediment examination (definitions–hematuria: ≥ 5 red blood cells per high power field (HPF); pyuria: ≥ 10 white blood cells per HPF^21,22^), randomization was performed by Kansai Medical University data center, with an online computer-generated system (mujinwari.biz/) using the dynamic allocation method, stratified by age (< 50 or ≥ 50 years), sex (male or female), and bacteria in urine (absent or present).

### Ureteral stent placement and removal

Ureteroscopy was performed under spinal or general anesthesia with a semi-rigid and/or flexible ureteroscope with or without a ureteral access sheath. After URS, a 6-Fr 26 cm ureteral stent (Polaris Ultra Ureteral Stent or Tria Ureteral Stent; Boston Scientific, Malborough, MA, USA) was appropriately inserted. Intraoperative antibiotics were used for all patients, and postoperative use depended on the physician’s discretion based on the patient’s condition. The stone-free status and stent location were evaluated by plain radiography on postoperative day (POD) 1. The patients were asked to call or visit the medical center regarding concerns about stent-related complications after discharge. On POD14 (− 2 or + 5 days allowed), we performed imaging for stent location and the degree of hydronephrosis by plain radiography and ultrasonography, respectively. The ureteral stent was removed using cystoscopy with grasping forceps.

### Sample processing and micro-CT evaluation

**Figure 3:**
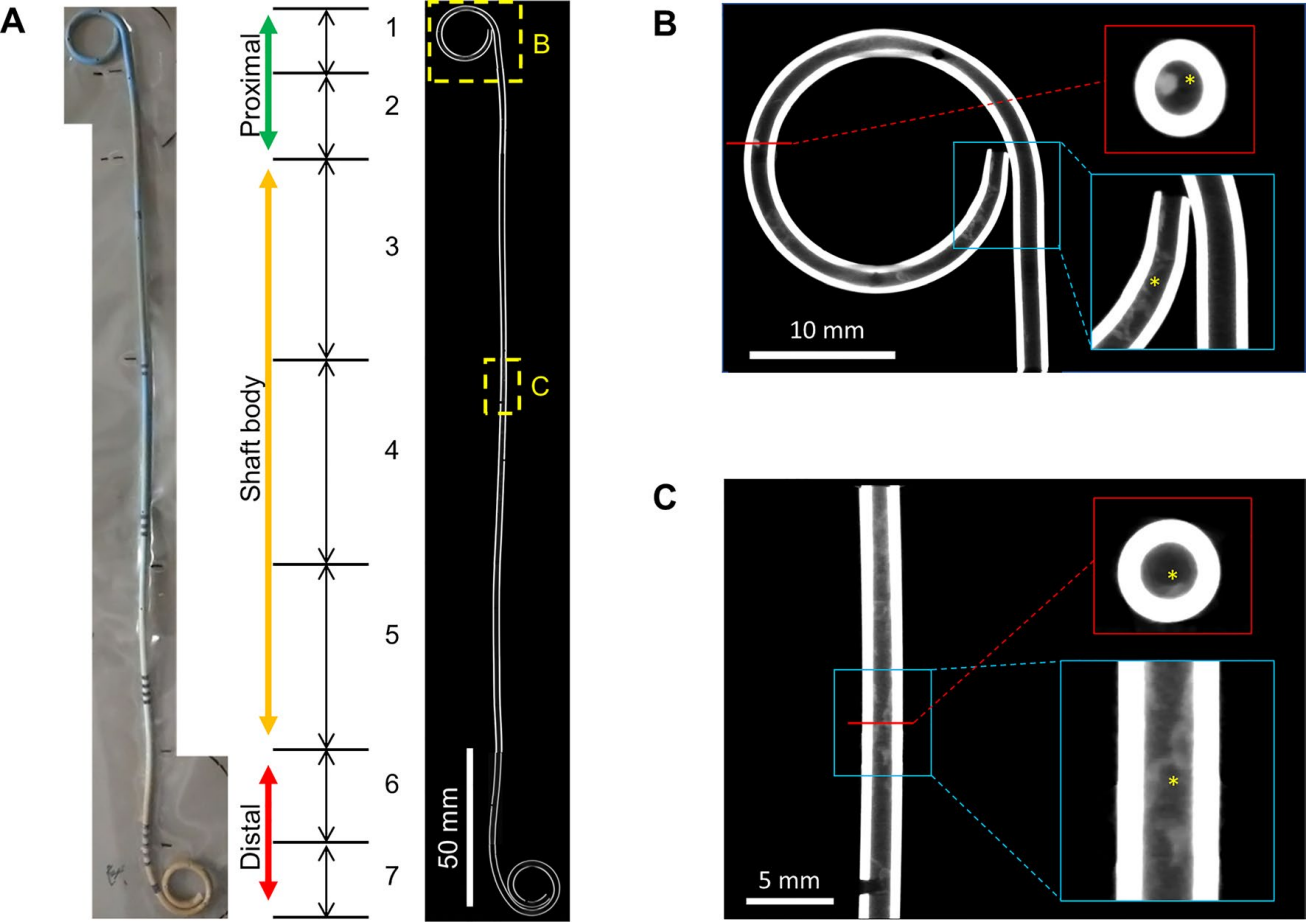
(**A**) Representative micro-computed tomography (micro-CT) imaging of the ureteral stent, combining the divided seven parts. The part surrounded by the dotted line shows a low power field in Fig. 2B and C. (**B**) High power view of the proximal loop. (**C**) High power view of the shaft body. The red line and blue-bordered box indicate axial and coronal (micro-CT) images, respectively. Asterisks indicate inner luminal encrustation.

An extracted ureteral stent was completely packed in a poly-nylon vacuum bag with about 1.0 g of silica gel using a vacuum apparatus, as the stent shaft straightened (Fig. 3A). All stent samples were collected at the Central Research of Laboratory. Micro-CT imaging for stents was acquired with full rotation and 360 projections using the Inveon small-animal single-photon emission CT/CT system (Siemens Medical Solutions USA, Inc., Malvern, PA) operated at 40 kVp and 500 μA with an exposure time of 3.5 s based on a preliminary experiment (Supplementary Fig. 2). The effective pixel size was 25.93 μm (maximum resolution of approximately 50 μm). The stent was separately imaged as seven sections due to the maximum axial field of view of 127 mm (Fig. 3A). Images were analyzed using ImageJ/Fiji software (NIH, version 1.52p, Bethesda, MD, USA)^22^ and MorphoLibJ, a collection of mathematical morphology methods and plugins for ImageJ, created at the INRA-IJPB Modeling and Digital Imaging lab^22,23^. Representative micro-CT images are demonstrated in Fig. 3A–C. Encrustation volume was calculated as follows: (1) A region of interest was fixed for each image; (2) After loading the dataset to Fiji, the images were converted to the 8-bit grayscale format; (3) “Plugin > MorphoLibJ > Morphological filters (3D)” tool was used to eliminate noises; (4) The stone segmentation and labeling was performed using “Plugin > MorphoLibJ > Segmentation > Morphological Segmentation” tool; (5) The encrustation volume was measured using “Plugin > MorphoLibJ > Analyze > Analyze Regions 3D” tool.

### Endpoints

The primary endpoint was the encrustation volume in the inner lumen of the stent. The secondary endpoints were the encrustation volume on the outer/total surfaces, partial encrustation volume in each portion of the stent (proximal loop/shaft body/distal loop; Fig. 3A), degree of postoperative hydronephrosis on POD14 before stent removal, and stent-related complications. We undertook a post-hoc analysis to assess the clinical factors associated with the total amount of encrustation.

### Statistical analysis

There have been few studies on the measurement of the total encrustation volume of the stent in humans. Therefore, the sample size was calculated based on public data from Boston Scientific—the in vitro experiment evaluating calcium/magnesium salt adhesion between the Tria ureteral stent versus the conventional hydro-coated ureteral stent with a mean value of 0.4856 mg/cm^2^ and 1.5705 mg/cm^2^, respectively (*P* < 0.05) (www.bostonscientific.com/jp-JP/products/stent/Tria.html). To detect a 1.0849 difference between the two stents, a standard deviation (SD) of 1.5, a 2-tailed significance level of 0.05, and a power of 0.80, we required sample size of 31 patients per arm. Therefore, 40 patients per group were adequate for statistical analysis, which allowed for a dropout rate of 20%.

The modified intention-to-treat (ITT) population, apart from those with no extracted ureteral stent, was used for the final analysis of endpoints. The chi-square test was used to compare nominal variables, and Wilcoxon signed- rank test was used to compare continuous variables between the two arms. Continuous data are expressed as median interquartile range (IQR). Pairwise multiple comparisons were performed by the Friedman test with the Bonferroni correction. Simple or multiple linear regression analysis was used to assess the association between the potential factor and encrustation volume. All statistical analyses were performed using EZR version 1.37 (Saitama Medical Center, Jichi, Japan)^24^. All reported values were two-sided with statistical significance set at 0.05.

## Supplementary Information

Supplementary Information.

## Abbreviations

HPF: High power field
Micro-CT: Micro-computed tomography
URS: Ureteroscopy
POD: Postoperative day
IQR: Interquartile range
ITT: Intention to treat
