# Supplementary material for: A randomized clinical trial evaluating the short-term results of ureteral stent encrustation in urolithiasis patients undergoing ureteroscopy: micro-computed tomography evaluation
Source: Sci Rep. 2021 May 14;11:10337. doi: 10.1038/s41598-021-89808-x (PMC8121799; doi:10.1038/s41598-021-89808-x)
Supplement: Supplementary file 1 — Supplementary Information. [file 41598_2021_89808_MOESM1_ESM.docx]

**A Randomized Clinical Trial Evaluating the Short-Term Results of Ureteral Stent Encrustation in Urolithiasis Patients Undergoing Ureteroscopy: Micro-Computed Tomography Evaluation**

Takashi Yoshida^1, 2^, Kuniko Takemoto^3^, Yoshiko Sakata^4^, Tomoaki Matsuzaki^1^, Yuya Koito^2^, Shimpei Yamashita^5^, Isao Hara^5^, Hidefumi Kinoshita^1^, and Tadashi Matsuda^1^

^1^Department of Urology and Andrology, Kansai Medical University, Osaka, Japan

^2^Department of Urology and Andrology, Kori Hospital, Kansai Medical University, Osaka, Japan

^3^Department of Physics, Kansai Medical University, Osaka, Japan

^4^Central Research of Laboratory, Kansai Medical University, Osaka, Japan

^5^Department of Urology, Wakayama Medical University, Wakayama, Japan.

Corresponding Author: Takashi Yoshida, MD

Department of Urology and Andrology, Kansai Medical University

2‐5‐1, Shin‐machi, Hirakata 573‐1152, Japan.

Tel: +81-72-804-0101; Fax: +81-72-804-2068

**Supplementary Fig. 1.** Relative abundance rate of extracted stone components in each patient.


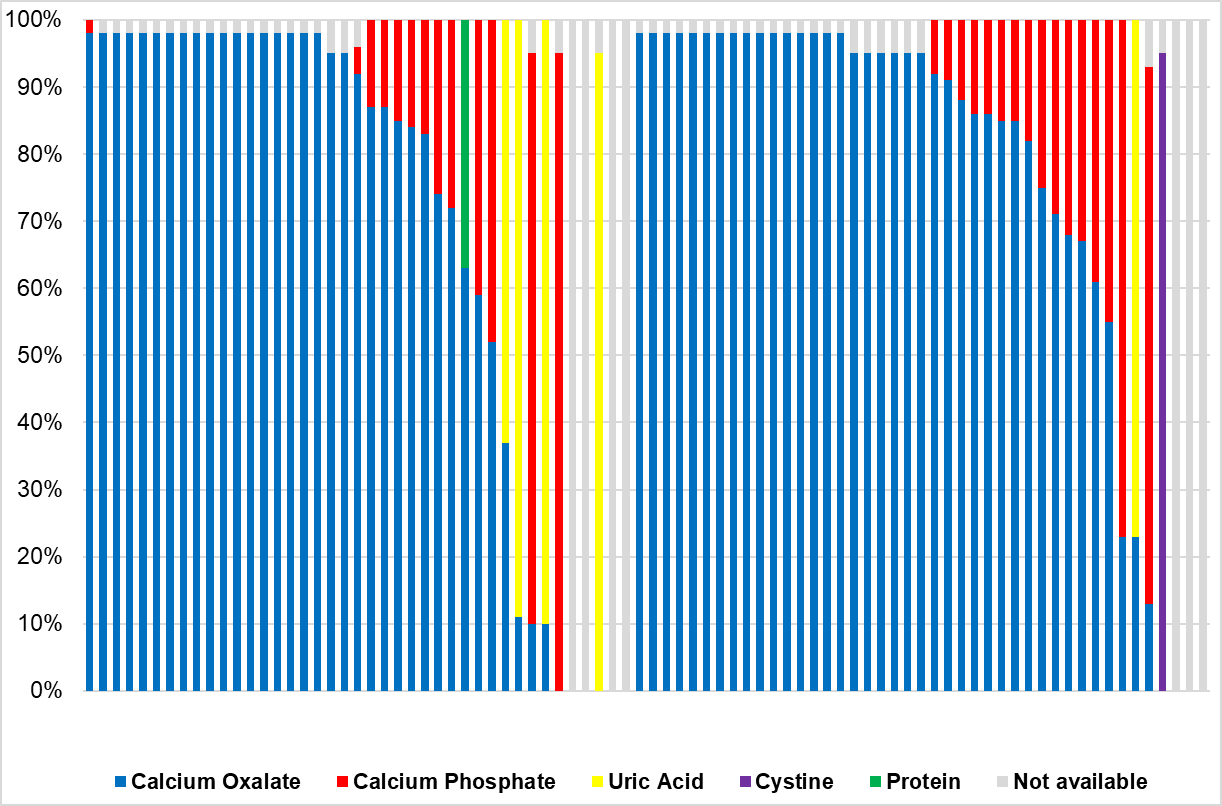


**Tria**

**Polaris Ultra**

**Relative abundance rate**

**Supplementary Fig. 2.** The computed tomography (CT) values of a stent and five main types of kidney stones under different photon energies. Except for uric acid, the CT values of kidney stones increase with decreasing energy. The CT value differences between the stent and the stones also increase with decreasing energy. When kidney stones are small, high CT values are favorable. Stent (PU)+5% Bi: 5 wt% bismuth-containing polyurethane stent, and MAP: magnesium ammonium phosphate.


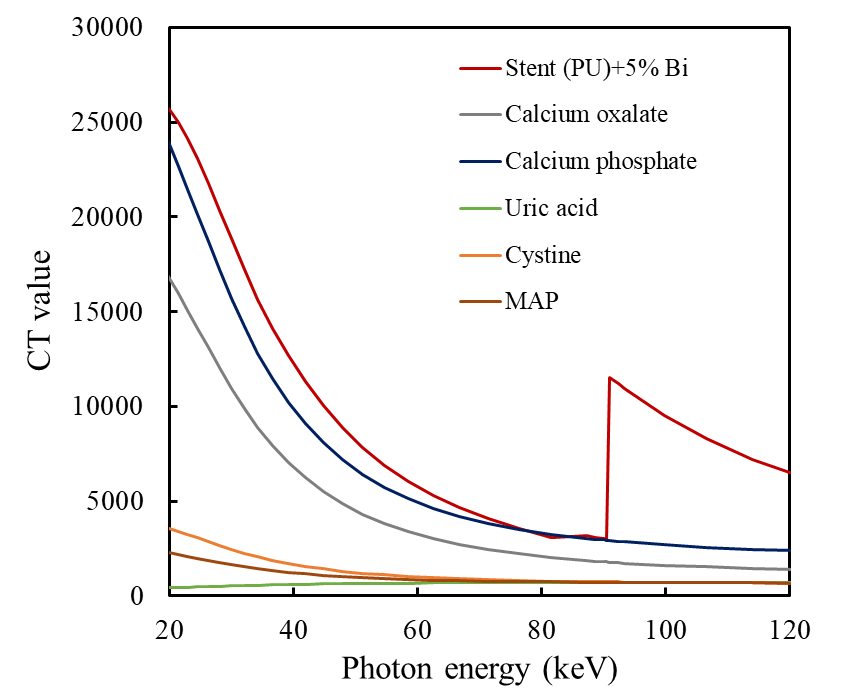


| **Supplementary Table 1. Uni- and multivariate analysis of preoperative clinical and stent-related factors associated with encrustation formation.** | | | | | | | | | | |
| --- | --- | --- | --- | --- | --- | --- | --- | --- | --- | --- |
|  | Univariate analysis | | | |  | Multivariate analysis | | | | |
|  | Β | SE | t statistic | P value |  | β | SE | t statistic | P-value |  |
| Age, year | -0.223 | 0.258 | -0.866 | 0.389 |  | -0.396 | 0.25018 | 2.259 | 0.117 |  |
| Sex (male vs. female) | 5.682 | 6.982 | 0.814 | 0.418 |  | 6.656 | 6.68233 | 0.996 | 0.322 |  |
| Body mass Index, kg/m^2^ | -0.129 | 0.832 | -0.154 | 0.878 |  |  |  |  |  |  |
| Hypertension (yes vs. no) | -4.628 | 7.034 | -0.658 | 0.513 |  |  |  |  |  |  |
| Dyslipidemia (yes vs. no) | 18.492 | 8.851 | 2.089 | 0.040 |  | 19.604 | 8.680 | 2.259 | 0.027 |  |
| Hyperuricemia (yes vs. no) | -4.089 | 10.441 | -0.392 | 0.696 |  |  |  |  |  |  |
| Diabetes mellitus (yes vs. no) | -5.162 | 12.226 | -0.422 | 0.674 |  |  |  |  |  |  |
| Urine pH | 10.431 | 5.791 | 1.801 | 0.075 |  | 11.454 | 5.654 | 2.026 | 0.046 |  |
| Hematuria (yes vs. no) | -0.023 | 7.260 | 0.616 | 0.540 |  |  |  |  |  |  |
| Pyuria (yes vs. no) | -7.484 | 6.807 | -1.099 | 0.274 |  |  |  |  |  |  |
| Bacteriuria (yes vs. no) | -5.132 | 8.417 | -0.610 | 0.544 |  |  |  |  |  |  |
| Crystalluria (yes vs. no) | 16.933 | 8.420 | 2.011 | 0.048 |  | 21.592 | 8.149 | 2.650 | 0.010 |  |
| Urinary culture (positive vs. other) | 3.8007 | 3.930 | 0.967 | 0.336 |  |  |  |  |  |  |
| Hydronephrosis |  |  |  |  |  |  |  |  |  |  |
| Grade 0 | Reference |  |  |  |  |  |  |  |  |  |
| Grade 1 | -10.805 | 10.233 | -1.056 | 0.294 |  |  |  |  |  |  |
| Grade 2 | -10.742 | 8.738 | -1.229 | 0.223 |  |  |  |  |  |  |
| Grade 3 | -10.569 | 10.233 | -1.033 | 0.305 |  |  |  |  |  |  |
| Grade 4 | -7.812 | 18.786 | -0.416 | 0.679 |  |  |  |  |  |  |
| Stone side (left vs. right) | -3.394 | 2.081 | -1.631 | 0.107 |  |  |  |  |  |  |
| Stone location |  |  |  |  |  |  |  |  |  |  |
| Distal ureter | Reference |  |  |  |  |  |  |  |  |  |
| Upper ureter | 0.656 | 10.652 | 0.062 | 0.951 |  |  |  |  |  |  |
| Middle ureter | -0.070 | 15.518 | -0.004 | 0.996 |  |  |  |  |  |  |
| Renal pelvis | 9.309 | 10.382 | 0.897 | 0.373 |  |  |  |  |  |  |
| Stone size, mm | 1.064 | 0.727 | 1.464 | 0.147 |  |  |  |  |  |  |
| Type of stent (Polaris Ultra vs. Tria) | -6.868 | 6.797 | -1.011 | 0.315 |  |  |  |  |  |  |
| Duration of stent placement, day | -0.468 | 0.603 | 0.776 | 0.440 |  |  |  |  |  |  |
| Single and multiple linear regression models were used for statistical analysis. SE, standard error. | | | | | | | | | | |
